# Unraveling the immunological landscape in acute pancreatitis progression to sepsis: insights from a Mendelian randomization study on immune cell traits

**DOI:** 10.3389/fimmu.2024.1374787

**Published:** 2024-03-27

**Authors:** Wenbin Liu, Xiaofeng Wang, Shanzhi Zhao, Song Yang, Xiangtao Zheng, Fangchen Gong, Lei Pei, Dan Xu, Ranran Li, Zhitao Yang, Enqiang Mao, Erzhen Chen, Ying Chen

**Affiliations:** ^1^ Department of Emergency, Ruijin Hospital, Shanghai Jiao Tong University School of Medicine, Shanghai, China; ^2^ Department of Critical Care Medicine, Ruijin Hospital, Shanghai Jiao Tong University School of Medicine, Shanghai, China

**Keywords:** acute pancreatitis, sepsis, immune cell traits, Mendelian randomization, CD127

## Abstract

**Background:**

Acute pancreatitis (AP) is a severe digestive system disorder with a significant risk of progressing to sepsis, a major cause of mortality. Unraveling the immunological pathways in AP is essential for developing effective treatments, particularly understanding the role of specific immune cell traits in this progression.

**Methods:**

Employing a bidirectional two-sample Mendelian Randomization (MR) approach, this study first examined the causal relationship between AP and 731 immune cell traits to identify those significantly associated with AP. Subsequently, we explored the causal associations between 731 immune cell traits and sepsis. The analysis utilized extensive genome-wide association studies (GWAS) summary datasets, with a focus on identifying common immune cell traits with statistically significant causal associations between AP and sepsis.

**Results:**

Our investigation identified 44 immune cell traits unidirectionally associated with AP and 36 traits unidirectionally associated with sepsis. Among these, CD127 on CD28^+^ CD45RA^-^ CD8^+^ T cells emerged as a common mediator, accounting for 5.296% of the increased risk of sepsis in AP patients. This finding highlights the significant role of specific memory CD8^+^ T cells in the pathophysiology of AP and its progression to sepsis.

**Conclusion:**

This study elucidates the critical role of specific immune cell traits, particularly CD127^hi^ memory CD8^+^ T cells, in the progression of AP to sepsis. Our findings provide a foundation for future research into targeted immune-modulatory therapies, potentially improving patient outcomes in AP-related sepsis and offering new insights into the complex immunological dynamics of this condition.

## 1 Introduction

Acute pancreatitis (AP), characterized by its acute onset within the digestive system, has historically posed challenges for effective treatment, leading to a poor prognosis, with approximately one-fifth of patients progressing to severe disease and a mortality rate of around 20% (1). One of the primary reasons for this unfavorable outcome is the development of sepsis secondary to pancreatitis, which is closely related to post-discharge mortality (2). In the course of pancreatitis, some patients may progressively develop into Infectious Pancreatic Necrosis (IPN), a severe infection state that may trigger the onset of sepsis (3). Microbial colonization of non-viable pancreatic tissue leads to infected necrosis, and this process is the primary factor contributing to the development of infectious complications and increased mortality (4). Additionally, pancreatitis can lead to extra-pancreatic infections, such as infectious pneumonia and unitary tract infection, which may develop to sepsis (5, 6).

The progression from AP to sepsis has been a focal point of recent research, seeking to understand the underlying mechanisms. Studies have identified key factors contributing to this progression, including a decrease in peripheral absolute lymphocyte count has been associated with a higher incidence of IPN (7). Furthermore, the downregulation of HLA-DR and alterations in the Kynurenine pathway, which have been linked to the onset of IPN during AP (8), and treatments like thymosin alpha 1, aimed at enhancing immunity, show potential in reducing the occurrence of IPN in AP patients (9). Additionally, factors such as intestinal barrier dysfunction, characterized by increased gut permeability and bacterial translocation, contribute to the severity of AP and the development of extra-pancreatic infections (6). The Compensatory Anti-Inflammatory Response Syndrome (CARS), associated with immunosuppression, can further exacerbate secondary infections in pancreatic necrosis, leading to sepsis-related complications (10).

The role of various immune cells in the development of AP is pivotal, as they act as key mediators potentially involved in the progression of pancreatitis to sepsis (11). Despite these advances, a comprehensive understanding of the transition from AP to sepsis, particularly the role of specific immune cell traits, remains elusive. Our study endeavors to explore the potential causal relationships involved in this progression by utilizing MR. The MR approach is predicated on the premise of causality (12); thus, we begin with the hypothesis that changes in the functionality of immune cells are instrumental in the advancement of AP to sepsis. MR studies, emerging in parallel with advancements in GWAS, provide a novel approach to establish causal relationships (13). MR studies are increasingly illuminating the factors associated with the pathogenesis and prognosis of AP (14). Our research is aimed to explore the complex interplay between immune cell traits and their impact on the progression from AP to sepsis. By applying MR, we aim to fill the current knowledge gap and add a layer of causal inference to the observed associations. The insights gained from our study could significantly enhance our comprehension of immune responses in AP and inform the creation of targeted therapeutic strategies, ultimately seeking to improve clinical outcomes for patients suffering from AP-induced sepsis.

## 2 Materials and methods

### 2.1 Data sources and research design

The MR analysis employed in this study is based on the premise that there is a causal connection between the functionality of immune cells and the progression to more severe disease states following AP. This study utilized extensive GWAS summary datasets, with participant consent obtained in the original studies, thus negating the need for additional ethical approval. We employed a bidirectional two-sample MR approach to explore the mutual causal relationship between AP and sepsis, focusing on the mediating role of immune cells. The AP dataset, publicly available and primarily of European ancestry, included genetic associations for 10,630 cases and 844,679 controls (15). Similarly, the sepsis dataset, also publicly accessible and predominantly of European ancestry, comprised 1,573 cases and 454,775 controls (16). Genetic associations for 731 complex immune cell traits from blood were sourced from the IEU Open GWAS project (17), with detailed identifiers listed in **Supplementary Table S1**.

### 2.2 Instrumental variable selection and data preparation

Instrumental variables (IVs) were identified using their respective GWAS IDs, retrieving SNP data including beta coefficients, standard errors, allele details, frequencies, p-values, and sample sizes. Stringent criteria ensured IVs met three key assumptions. We adjusted our SNP selection threshold to p < 5 × 10^-5^ for AP, sepsis, and immune cell data. Linkage disequilibrium clustering was performed using a 10,000 kb window and an r^2^ threshold of < 0.001, utilizing PLINK software (https://www.cog-genomics.org/plink2/). Palindromic or ambiguous SNPs were excluded.

### 2.3 Data harmonization and MR estimation

Data harmonization ensured consistency in effect direction and allele coding across SNPs. The instrumental strength of each SNP was assessed using R² and F-statistics, excluding SNPs with an F-statistic below 10 (**Supplementary Table S2**). MR estimates, including Odds Ratios (ORs) and p-values, were calculated for each exposure factor to identify significant associations (p < 0.05). We employed the Inverse Variance Weighting (IVW) method for primary estimation, supplemented by MR-Egger and the Weighted Median approach, each tailored to specific instrumental variable validity assumptions.

### 2.4 Sensitivity analyses

MR Steiger filtering was applied to determine the causal direction of each SNP relative to the exposure and outcome. This method compared the variance explained in the exposure by instrumental SNPs against that in the outcome. ‘TRUE’ outcomes indicated causality in the expected direction, while ‘FALSE’ results suggested the opposite. SNPs with ‘FALSE’ outcomes, predominantly influencing the outcome, were discarded. SNP homogeneity was assessed using Cochran’s Q statistic and funnel plots. Horizontal pleiotropy was examined via MR-Egger intercept and MR-PRESSO methods, with outliers being removed for a re-evaluation of MR causal inferences. Persistent heterogeneity necessitated the use of a random effects model. Finally, a leave-one-out analysis was conducted for individual SNP impact assessment.

### 2.5 Selection of immune cells

A two-sample analysis assessed the mutual causality between AP and sepsis, followed by AP and immune cells, as well as immune cells and sepsis. Statistically significant associations (p < 0.05) were identified using the IVW method. A Venn diagram was utilized to identify common immune cells with statistically significant causal associations, which were then selected as intermediate factors to investigate their connection from AP to sepsis.

### 2.6 Mediation analysis of intermediate effect

**Figure 1:**
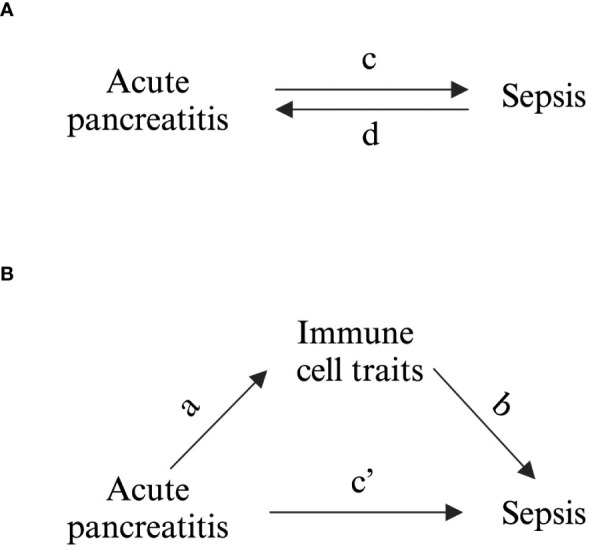
Schematic Representation of Study Associations. **(A)** Illustration of the overall impact, with c denoting the influence of genetically inferred AP on sepsis, and d indicating the influence of genetically inferred sepsis on AP. **(B)** Breakdown of the overall impact into (i) the indirect impact (a × b, where a represents the influence of AP on immune cell characteristics and b represents the impact of these characteristics on sepsis) and (ii) the direct impact (c′ = c – a × b). The mediated proportion is determined by dividing the indirect impact by the overall impact.

The total effect, representing bidirectional MR between AP and sepsis, was initially designated (**Figure 1A**). A two-step bidirectional MR design then facilitated mediation analysis to examine if immune cell traits mediate the progression from AP to sepsis (**Figure 1B**). The total effect was decomposed into direct effects (c’ in **Figure 1B**) and indirect effects mediated via immune cell traits (a × b in **Figure 1B**). The mediation percentage was calculated by dividing the indirect effect by the total effect.

## 3 Result

### 3.1 Causal relationship between acute pancreatitis and sepsis

**Figure 2:**
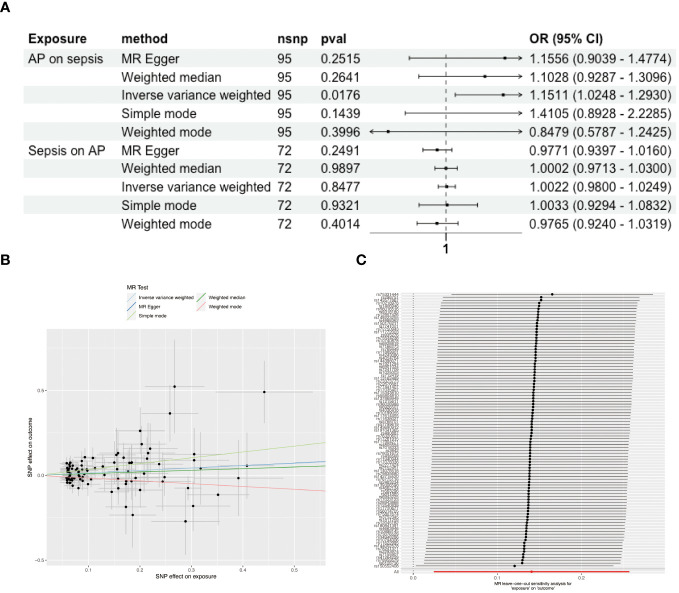
Causal Associations Between AP and Sepsis. **(A)** Forest plot visualizing the causal effects of AP on sepsis and sepsis on AP. **(B)** Scatter plot displaying the positive correlation between AP and sepsis risk across different SNPs. **(C)** Leave-one-out plot illustrating the causal effect stability when excluding one SNP at a time.

After excluding palindromic, ambiguous SNPs, and those with incorrect causal directions as per MR Steiger filtering, we identified 95 SNPs associated with AP and 74 SNPs linked to sepsis as instrumental variables (**Supplementary Tables S2**). Forest plots illustrating each AP SNP’s relationship with sepsis are in **Supplementary Figure S1**. Using the IVW method, complemented by MR Egger, weighted median, and mode approaches, we found positive causal association of AP on sepsis (OR = 1.151, 95% CI = 1.025-1.293) (**Figure 2A**), with upward trends in plots supporting positive correlations (**Figure 2B**). Reverse MR showed no causal link from sepsis to AP (**Figure 2A**). Sensitivity analyses indicated no significant heterogeneity among SNPs (**Supplementary Table S3**), and the MR-Egger intercept test suggested no pleiotropy (**Supplementary Table S4**). Systematic re-analysis post-SNP removal confirmed these findings (**Figure 2C**).

### 3.2 Association of AP with 731 immune cell traits

**Figure 3:**
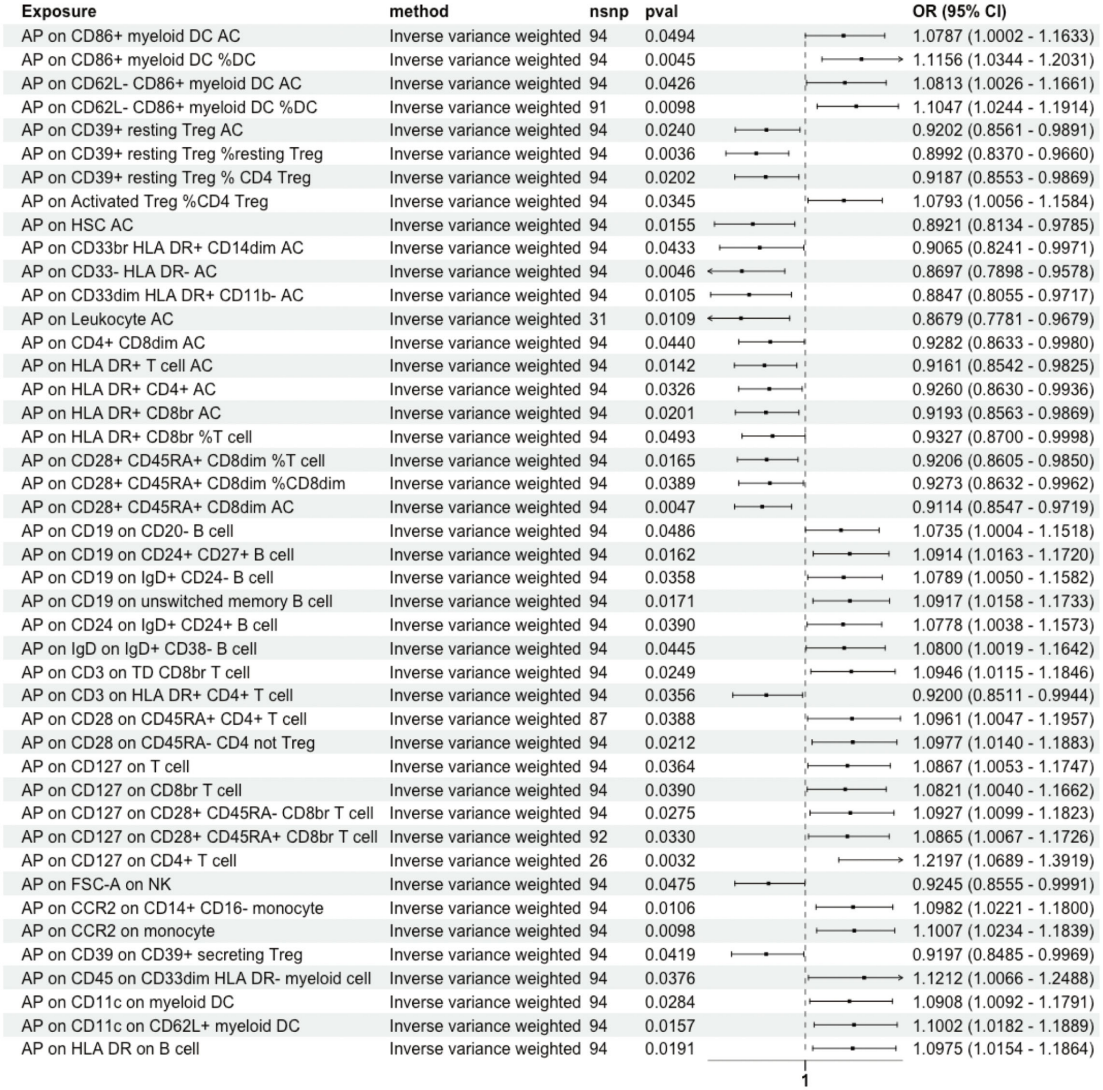
Unidirectional Causal Relationship Between AP and Immune Cell Traits. A unidirectional causal relationship was established between AP and the remaining 44 immune cell traits. DC, Dendritic Cell; AC, absolute count; HSC, Hematopoietic Stem Cell; Im, immature; MDSC, Myeloid-Derived Suppressor Cells; dim, diminished; br, bright; TD, terminally differentiated; NK, natural killer.

The influence of AP on 731 immune cell traits was assessed primarily using the IVW method. This analysis identified significant associations between AP and 50 immune cell traits. MR-Egger intercept test detected pleiotropy between AP and immature Myeloid-Derived Suppressor Cells absolute count (AC), CD127 on CD28^-^ CD8^br^ (**Supplementary Table S4**), while no SNP heterogeneity was observed between AP and these 50 immune cell traits (**Supplementary Table S3**). 25 immune cell traits showed significant causal associations with AP. Notably, HLA DR^+^ NK AC, CD19 on IgD^+^ CD24^+^ B cells, CD19 on memory B cells, and IgD on transitional cells demonstrated bidirectional MR causal associations with AP (**Supplementary Figure S2**). The MR-Egger intercept test revealed pleiotropy between CD19 on memory B cells and AP, with no SNP heterogeneity detected for these 25 immune cell traits and AP (**Supplementary Tables S3, S4**). Consequently, a unidirectional causal relationship was established between AP and the remaining 44 immune cell traits (**Figure 3**).

### 3.3 Association of 731 immune cell traits with sepsis

**Figure 4:**
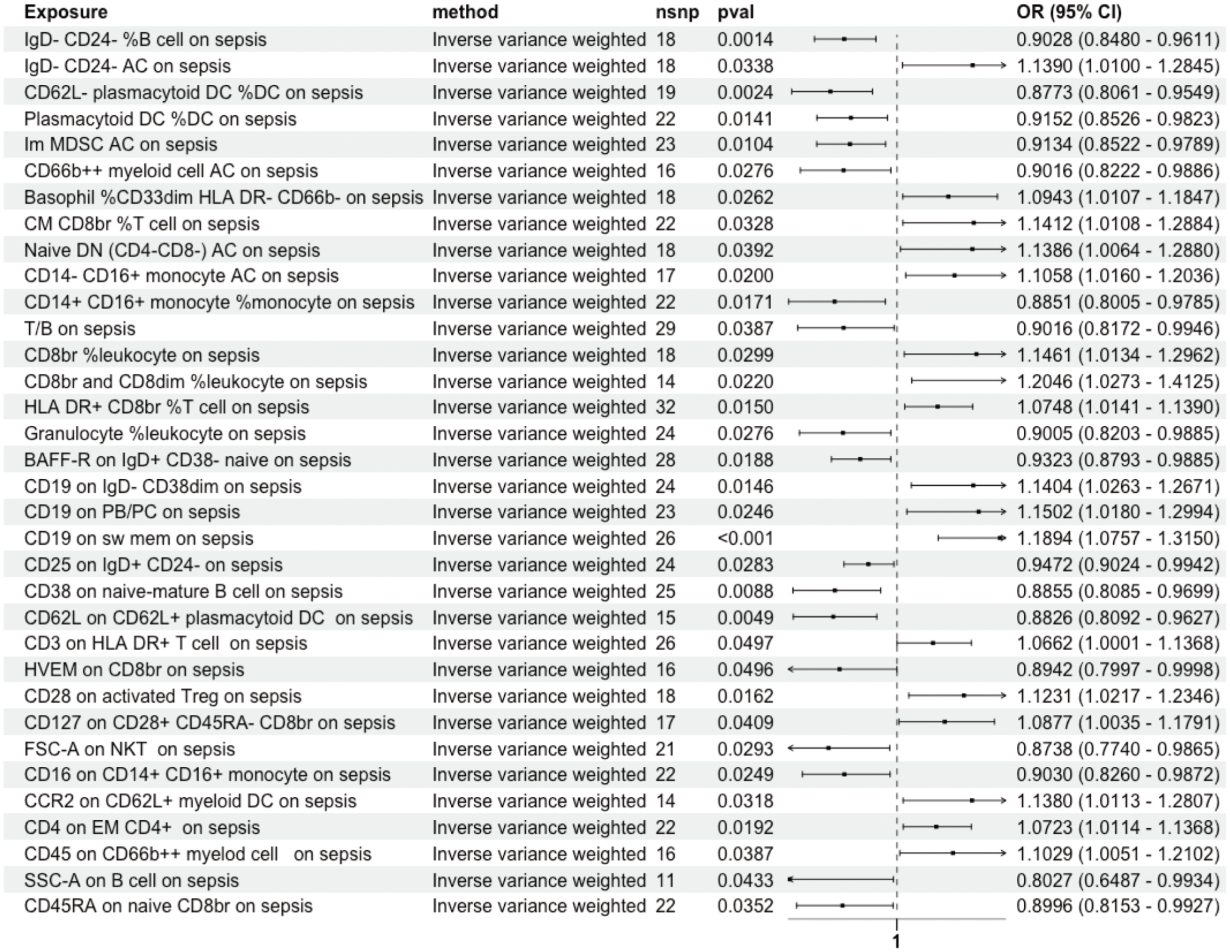
Unidirectional Causal Relationship Between Immune Cell Traits and Sepsis. A unidirectional causal relationship was established between 34 immune cell traits and sepsis. Im, immature; MDSC, Myeloid-Derived Suppressor Cells; AC, absolute count; dim, diminished; CM, Central Memory; br, bright; DN, double negative; PB/PC, Plasmablasts/Plasma Cells; sw mem, Switched Memory B Cells; EM, Effector Memory.

The influence of these immune cell traits on sepsis was investigated using the IVW method, revealing notable associations with 36 immune cell traits. MR-PRESSO indicated pleiotropy between CD3 on CD39^+^ activated Treg and sepsis (**Supplementary Table S4**), with no SNP heterogeneity found between these 36 traits and sepsis (**Supplementary Table S3**). sepsis was significantly causally associated with 21 immune cell traits. Among these, terminal differentiation (TD) CD4^+^ %T cells demonstrated bidirectional MR causal associations with sepsis (**Supplementary Figure S2**). The MR-Egger intercept test detected no pleiotropy, and no SNP heterogeneity was observed between sepsis and these 21 immune cell traits (**Supplementary Tables S3, S4**). As a result, a unidirectional causal relationship was established between the remaining 34 immune cell traits and sepsis (**Figure 4**).

### 3.4 Mediating role of immune cell traits in AP progression to sepsis

**Figure 5:**
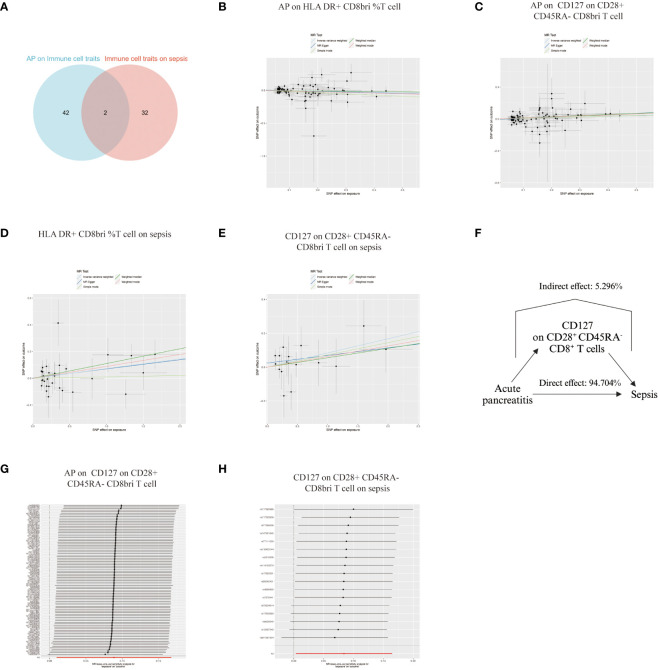
Analysis of Immune Cell Trait Associations with Acute Pancreatitis and Sepsis. **(A)** Venn diagram illustrating the overlap of immune cell traits associated with both AP and sepsis, highlighting HLA DR^+^ CD8^br^ %T cells and CD127 on CD28^+^ CD45RA^-^ CD8^br^ T cells as common links. **(B)** Plot showing a negative causal relationship between AP and the levels of HLA DR^+^ CD8^br^ %T cells. **(C)** Plot illustrating a positive causal relationship between AP and the levels of CD127 on CD28^+^ CD45RA^-^ CD8^br^ T cells. **(D, E)** Plots demonstrating the positive causal associations of HLA DR^+^ CD8^br^ %T cells and CD127 on CD28^+^ CD45RA^-^ CD8^br^ T cells with sepsis. **(F)** Graph indicating that CD127 on CD28^+^ CD45RA^-^ CD8^br^ T cells are responsible for 5.296% of the increased risk of sepsis in patients with AP. **(G, H)** Leave-one-out plot of a systematic re-analysis after SNP removal, confirming the stability and reliability of the associations.

Our analysis used Venn diagrams to identify key associations between immune cell traits, AP, and sepsis. HLA DR^+^ CD8^br^ %T cells and CD127 on CD28^+^ CD45RA^-^ CD8^br^ T cells were linked not only to AP as an outcome but also as exposures in relation to sepsis (**Figure 5A**). AP demonstrated a negative causal association with levels of HLA DR^+^ CD8^br^ %T cells (**Figure 5B**) and a positive causal association with CD127 on CD28^+^ CD45RA^-^ CD8^br^ T cells (**Figure 5C**). Both cell types had positive causal associations with sepsis (**Figures 5D, E**). However, the opposing directions of Mendelian causal associations for HLA DR^+^ CD8^br^ %T cells in AP and sepsis precluded its role as correlated mediator. In contrast, CD127 on CD28^+^ CD45RA^-^ CD8^br^ T cell accounted for 5.296% of the increased risk of AP for sepsis (**Figure 5F**). The absence of heterogeneity and pleiotropy was confirmed, and systematic re-analysis post-SNP removal validated these significant causal relationships (**Supplementary Tables S3, S4**, **Figures 5G–H**).

## 4 Discussion

The pathophysiological mechanisms underlying severe acute pancreatitis and sepsis exhibit notable similarities, particularly in terms of dysregulated immune responses that lead to life-threatening organ dysfunction (18, 19). The primary distinction between these conditions lies in their initial stimuli: pancreatitis is typically a response to biochemical stimuli from leaked pancreatic fluids, whereas sepsis arises from inflammatory responses to pathogenic infections. Despite these insights, the detailed mechanisms by which AP progresses to sepsis remain elusive. Our study leverages MR to probe into the intricate relationship between AP, immune cell traits, and sepsis pathogenesis. By using genetic variants as instrumental variables, MR helps infer causal relationships between these elements, offering a robust framework that is less susceptible to the biases of reverse causation and confounding factors commonly encountered in traditional observational studies.

In our analysis, we established a unidirectional causal association between AP and 44 immune cell traits, and similarly, 34 immune cell traits showed a unidirectional causal association with sepsis. Notably, HLA DR^+^ CD8^br^ %T cells and CD127 on CD28^+^ CD45RA^-^ CD8^br^ T cells emerged as common immune cell traits, serving as outcomes in AP and exposures in sepsis. Specifically, CD127 on CD28^+^ CD45RA^-^ CD8^br^ T cells accounted for 5.296% of the increased risk of AP progressing to sepsis. CD8, a co-receptor molecule predominantly found on cytotoxic T lymphocytes (CTLs), plays a crucial role in immune responses (20). CD28 is a co-stimulatory molecule, and its expression indicates that T cells can receive immune activation signals, and according to CD28 positive or negative CD8^+^ cells can be divided into cytotoxic T cells (CD28^+^) and inhibitory T cells (CD28^-^) (21). CD45RA, often linked with naïve T cells, denotes cells yet to encounter their specific antigen (22). Based on the above information, CD28^+^ CD45RA^-^ CD8^br^ T cells typically represent mature, activated cytotoxic T cells with potential memory or effector properties. CD127, encoded by the IL7R gene, forming a heterodimeric IL-7 receptor with a common gamma-chain, is expressed across various T cell subsets throughout their lifecycle, regulating maturation, maintenance, and immune responses (23). The expression level of CD127 on CD8^+^ T cells often indicates their activation and memory status, with CD127^hi^ cells inclined towards prolonged survival and memory functions, whereas CD127^dim^ cells are more associated with activation or effector states (24). CD127 expression plays a pivotal role in the development of memory cells. Memory T cells can be further categorized into effector memory T cells (TEM), central memory T cells (TCM), peripheral memory T cells (TPM), tissue-resident memory cells (TRM), and terminally differentiated effector memory cells (TEMRA), among others, with TCM, TEM, TRM, and TPM typically expressing high levels of CD127 (25).

Our MR analysis revealed a positive causal association between the incidence of AP and the expression of CD127 on CD28^+^ CD45RA^-^ CD8^br^ T cells. Based on CD127 expression, CD8^+^ T cells can be classified into effector and memory T cells, suggesting a positive correlation between AP and the relative increase in memory CD8^+^ T cells/relative decrease in effector CD8^+^ T cells. Research on CD8^+^ T cells and memory T cells in the context of AP is notably scarce. Early reports indicate a reduction in CD8^+^ T cells during the initial stages of AP, yet without delving into specific CD8^+^ T cell subtypes (26). High expression of CD127, functioning as the receptor for IL-7, is crucial for the survival of functional memory CD8^+^ T cells. IL-7 plays a pivotal role in suppressing apoptosis, primarily through the upregulation of the anti-apoptotic protein BCL-2 and the downregulation of the pro-apoptotic protein Bax, which contributes to the maintenance and longevity of memory CD8^+^ T cells (27). In the early stages of AP, there is likely a substantial generation of effector CD8^+^ T cells. However, these effector cells often lack the protective effects conferred by CD127 expression. Without this protection, a significant proportion of these cells may enter apoptosis following their effector phase. This phenomenon could, to a certain extent, explain why AP leads to an increase in memory CD8^+^ T cells and a relative decrease in effector CD8^+^ T cells. The absence of CD127-mediated survival signals in effector CD8^+^ T cells during AP potentially accelerates their apoptosis post-activation. Consequently, this results in a relative enrichment of memory CD8^+^ T cells, which are better equipped to survive long-term due to their higher CD127 expression. The scarcity of research on memory T cells in AP contrasts with studies indicating that in chronic pancreatitis, macrophage-derived co-stimulation can enhance the effector functions of TRM, thereby promoting inflammatory progression (28). A similar interaction between macrophages and TRM cells in AP could potentially exacerbate inflammation.

The positive causal relationship between CD127 on CD28^+^ CD45RA^-^ CD8^br^ T cells and the onset of sepsis suggests that memory CD8^+^ T cells play a significant role in the progress of sepsis. Research involving mice with prior immune exposure has revealed a significant and varied population of memory T cells, both circulating in the bloodstream and residing in solid organs (29). One study demonstrated that immune education modifies CLP-induced changes in both innate and adaptive immunity, indicating that memory T cells contribute to “immune dysregulation” (30). Mice pre-exposed to immune stimuli possess an increased number of CD8^+^ effector memory T cells, leading to more severe skin inflammatory responses compared to unstimulated mice (31). The frequencies of CD8^+^ TCM positively correlate with the degree of steatosis, lobular inflammation, and ballooning in non-alcoholic fatty liver disease (32). CD8^+^ TCM cells in the brain can trigger inflammation by reactivating and expanding in response to signals from glial cells, which leads to an influx of other immune cells and increased local microglial activity, intensifying the immune response (33). CD8^+^ TRM cells respond to viruses by releasing important cytokines like IFN-γ and killing infected cells using perforin and granzyme B, especially when boosted by IL15 (34). Together with it, high level of memory CD8^+^ T cell might boost immune response and lead to dysregulation of immune, thus causing sepsis.

While our findings highlight the significance of CD127 expression on CD8+ T cells in the progression of AP to sepsis, it is crucial to consider the broader context of immune modulation in septic conditions. Interventional studies have shed light on the therapeutic potential of IL-7, the ligand for CD127, in reversing sepsis-induced lymphocytopenia (35). Moreover, innovative approaches to augment IL-7 in immunosuppressed septic shock patients have been shown to enhance T lymphocyte functions ex vivo, including increased production of IFN-γ and TNF-α (36). The ability of IL-7 to promote T cell viability, trafficking, and functionality aligns with observed improvements in survival rates among sepsis patients (37). This apparent contradiction, wherein CD127-mediated pathways contribute to the progression of sepsis while IL-7 supplementation shows therapeutic benefits, may be explained by considering the temporal dynamics of sepsis. The initial phase of sepsis is characterized by an excessive inflammatory response, with the progression from AP to sepsis largely attributed to this early hyperinflammatory phase. In contrast, the interventions involving IL-7 largely target the later stages of sepsis, which are marked by immunosuppression. In this phase, the supplementation of IL-7 can indeed mitigate the adverse outcomes by counteracting the immune suppression. Further research is needed to elucidate the intricate interplay between the signaling pathways mediated by CD127 and the therapeutic application of IL-7, particularly in delineating the mechanisms that govern the transition from hyperinflammation to immunosuppression in sepsis.

In our study, the contrasting causal relationships of HLA DR^+^ CD8^br^ %T cells in AP and sepsis suggest that rather than mediating the progression from AP to sepsis, they might actually serve as a protective factor against this development. CD8^+^ T cells, characterized by HLA-DR expression, are known for their potent capabilities including rapid proliferation, effective cytotoxic actions, and significant cytokine secretion (38). Nonetheless, post-functional completion, these cells are more prone to undergo cell death (39). In AP, the negative causal association with HLA DR^+^ CD8^br^ %T cell might be attributed to the activation of these cells by AP, but their inherent fragility leads to cell death, ultimately resulting in a negative correlation with AP. A high level of HLA DR^+^ CD8^br^ %T cells might indicate overactive inflammation, potentially leading to immune dysregulation and the onset of sepsis.

However, our study acknowledges several limitations inherent in the MR approach. Although MR significantly mitigates confounding, it cannot entirely preclude the potential for residual confounding, especially from factors not captured by the genetic instruments or from environmental factors interacting with genetic predispositions. Additionally, the reliance on GWAS summary datasets predominantly of European ancestry may not fully capture the genetic diversity of other populations, potentially limiting the generalizability of our findings. Therefore, future research utilizing individual-level data and including diverse populations is essential to validate and extend our insights. Furthermore, the results of our MR analysis are interpreted under the assumption that the functional changes in immune cells have a causal role in the development of sepsis from AP. It is important to consider the implications of this assumption on our findings. While the assumption is supported by existing literature, we must also acknowledge that if this causal relationship does not hold, it could limit the applicability of our conclusions. Future research may further validate this assumption and explore the complex interactions between immune cells and disease progression from AP to sepsis.

Our study opens a new avenue for the potential clinical application of therapeutic agents targeting immune cell pathways implicated in the progression of AP to sepsis. A case in point is GSK2618960, a humanized deactivating Fc monoclonal antibody that binds to the alpha-chain of the IL-7 receptor, impeding IL-7 binding and subsequent intracellular signaling. The safety of GSK2618960 has been preliminarily established in a Phase I clinical trial (I7R116702), targeting CD127 for autoimmune conditions, where it showed no significant adverse reactions in healthy volunteers. The demonstrated safety profile of GSK2618960 underscores its potential as a therapeutic option, although the current lack of clinical research on its efficacy in treating diseases necessitates further investigation (40, 41). Specifically, the feasibility of using GSK2618960 to prevent the progression of AP to sepsis remains an open question that our study’s findings render worthy of exploration. Future research should focus on this and other similar therapeutic strategies to ascertain their effectiveness in clinical settings and potentially improve outcomes for patients with AP at risk of developing sepsis.

## 5 Conclusion

Our study, employing Mendelian Randomization, reveals a significant causal relationship between acute pancreatitis and specific immune cell traits, particularly highlighting that CD127 on CD28^+^ CD45RA^-^ CD8^br^ T cells contributes to 5.296% of the increased risk of sepsis in AP patients. This finding emphasizes the crucial role of memory CD8^+^ T cells in the progression of AP to sepsis and opens new avenues for targeted immune-modulatory therapies. Our insights into the immune dynamics of AP provide a foundation for future research aimed at improving treatment strategies and patient outcomes in AP-related sepsis.

## Data availability statement

The original contributions presented in the study are included in the article/**Supplementary Material**. Further inquiries can be directed to the corresponding authors.

## Author contributions

WL: Formal analysis, Methodology, Software, Writing – original draft. XW: Methodology, Writing – review & editing. SZ: Methodology, Writing – review & editing. SY: Writing – review & editing. XZ: Visualization, Writing – review & editing. FG: Writing – review & editing. LP: Writing – review & editing. DX: Investigation, Writing – review & editing. RL: Investigation, Writing – review & editing. ZY: Validation, Writing – review & editing. EM: Validation, Writing – review & editing. EC: Funding acquisition, Supervision, Writing – review & editing. YC: Conceptualization, Supervision, Writing – review & editing.
