# Supplementary figures and images for: Unraveling the immunological landscape in acute pancreatitis progression to sepsis: insights from a Mendelian randomization study on immune cell traits
Source: Front Immunol. 2024 Mar 27;15:1374787. doi: 10.3389/fimmu.2024.1374787 (PMC11004341; doi:10.3389/fimmu.2024.1374787)

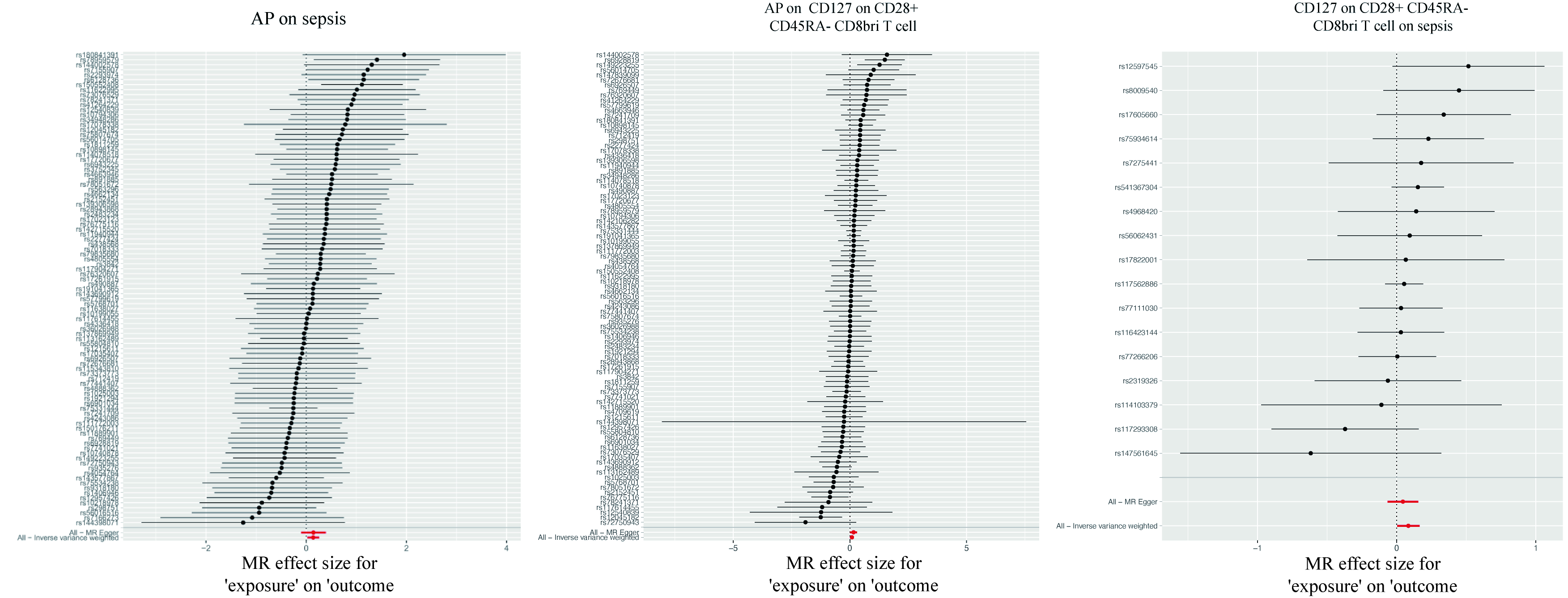

Supplement: Supplementary file 1 [file Image_1.tif]

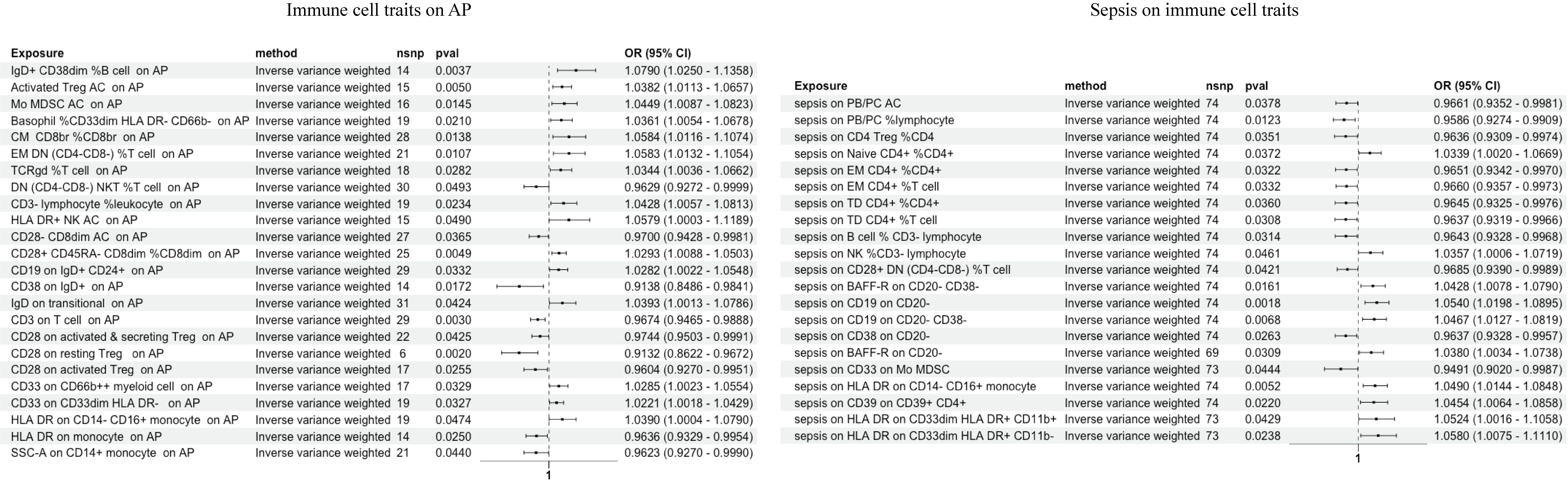

Supplement: Supplementary file 2 [file Image_2.png]
